# Phenolic Compounds and the Anti-Atherogenic Effect of Bee Bread in High-Fat Diet-Induced Obese Rats

**DOI:** 10.3390/antiox9010033

**Published:** 2019-12-30

**Authors:** Zaidatul Akmal Othman, Wan Syaheedah Wan Ghazali, Liza Noordin, Nurul Aiman Mohd. Yusof, Mahaneem Mohamed

**Affiliations:** 1 Department of Physiology, School of Medical Sciences, Universiti Sains Malaysia, Kubang Kerian 16150, Kelantan, Malaysia; zaidaakmal@unisza.edu.my (Z.A.O.); syaheeda@usm.my (W.S.W.G.); lizakck@usm.my (L.N.); 2 Faculty of Medicine, Universiti Sultan Zainal Abidin, Kuala Terengganu 20400, Terengganu, Malaysia; 3 Department of Anatomy, School of Medical Sciences, Universiti Sains Malaysia, Kubang Kerian 16150, Kelantan, Malaysia; aimannur@usm.my; 4 Unit of Integrative Medicine, School of Medical Sciences, Universiti Sains Malaysia, Kubang Kerian 16150, Kelantan, Malaysia

**Keywords:** bee bread, obese, anti-atherogenic, phenolic compounds

## Abstract

This study was undertaken to determine the phenolic compounds and the anti-atherogenic effect of bee bread in high-fat diet (HFD)-induced obese rats. The presence of phenolic compounds in bee bread was determined by liquid chromatography–mass spectrometry. Thirty-two male Sprague Dawley rats were divided into four groups, (*n* = 8/group); i.e., Normal (N), HFD (high-fat diet), HFD + BB (high-fat diet and 0.5 g/kg/day bee bread), and HFD + O (high-fat diet and 10 mg/kg/day orlistat) groups. After 6 weeks of the experiment, rats were sacrificed. Five phenolic compounds were identified in bee bread; namely, caffeic acid, ferulic acid, kaempferol, apigenin, and isorhamnetin. Bee bread significantly reduced Lee obesity index and levels of total cholesterol (TC), low-density lipoprotein (LDL), fatty acid synthase (FAS) activity, atherogenic index, oxidised-LDL (oxLDL), and malondialdehyde (MDA), and significantly increased aortic antioxidant activities, such as those of superoxide dismutase (SOD) and glutathione peroxidase (GPx). Adipocyte sizes were found to be smaller in the HFD + BB group compared to the N group, and en face aortas showed an absence of atherosclerotic plaque in rats supplemented with bee bread. These changes might suggest an anti-atherogenic effect of bee bread in HFD-induced obese rats via its antioxidant and hypocholesterolaemic properties.

## 1. Introduction

Obesity is a major contributor to total burden of disease in developing countries. Obesity has been an important target for health professionals to reduce obesity-related cardiovascular disease (CVD), notably dyslipidaemia, hypertension, and coronary artery disease [1]. Prolonged administration of a high-fat diet (HFD) to rats has been shown to develop a status of impaired-lipid metabolism, as evidenced by higher levels of total cholesterol (TC), triglyceride (TG), and low-density lipoprotein (LDL) in rats fed with a HFD [2]. Apart from impairment of lipid metabolism, attention has also recently focused on the role of low grade chronic inflammation as one of the mechanisms of obesity related disorders. It has been speculated that an excess of adipose tissue accumulation is a precursor of pro-inflammatory cytokines’ production contributing to adverse obesity-related complications, such as insulin resistance and increased blood pressure [3].

Recent decades have shown that there is increasing interest to assess the potential medicinal and therapeutic properties of natural products to prevent obesity-related CVD [4,5]. The naturally occurring products have been shown to possess anti-atherogenic effects by improving lipid profile, reducing oxidative stress status, and increasing the antioxidant enzyme defence mechanism [6,7]. Bee bread is one of the bee kingdom products formed by bees. It contains a mixture of bee pollen and bees’ digestive enzymes, and is abundantly found in the beehive. After 2 weeks, anaerobic lactic acid fermentation process contributes to a greater nutritive value of bee bread [8]. Bee bread is a well-balanced diet, as it contains carbohydrate, lipid, and protein sources, and essential minerals and vitamins, such as vitamins C, B1, B2, E, iron, calcium, and magnesium [9]. It also contains phenolic compounds, such as p-coumaric acid, kaempherol, isorhamnetin [10], apigenin, chrysin, ferulic acid, caffeic acid, gallic acid, naringenin, and quercetin [11,12]. Bee bread has been shown to offer protection against tumour cells reactivity and possess other biological properties, such as antimicrobial and hepatoprotective effects [11,13,14]. Moreover, it has been shown to exert a hypocholesterolaemic property by significantly reducing 15.7% of TC and 20.5% LDL levels in overweight and obese patients [15]. Studies of antioxidant activities of bee bread have been reported in few countries, including Lithuania [16], Araucania [17], Poland [11], Ukraine [18], Romania [19], and Georgia [20]. However, no study has been reported on its possible anti-atherogenic effect in obesity so far. Hence, the present study was aimed to determine the phenolic compounds’ presence in Malaysian bee bread and its anti-atherogenic effect in HFD-induced obese rats.

## 2. Materials and Methods 

### 2.1. Materials

Animal ghee was purchased from Unilever Holdings Sdn. Bhd. (Kuala Lumpur, Malaysia). Calcium and vitamin D were purchased from Eurobio Sdn. Bhd. (Victoria, Australia). Orlistat was purchased from Xepa-Soul Pattinson Sdn. Bhd. (Melaka, Malaysia), cholesterol powder from Nacalai Tesque (Kyoto, Japan), Eosin Y from Sigma-Aldrich (St. Louis, MI, USA), haematoxylin from Richard-Allan Scientific (Kalamazoo, MN, USA), and oil-Red O from VWR Life-Science AMRESCO (Solon, OH, USA). All other reagents were of analytical grade. 

### 2.2. Preparation of Bee Bread Sample

Bee bread from stingless bee (*Heterotrigona itama*) was purchased from local stingless bee farm from Selangor, Malaysia. The sample was dried using food dehydrator at 35 °C. Then, it was ground into fine powder using a mini blender and kept in a sterilised container at –20 °C until further analysis.

### 2.3. Liquid-Chromatography-Mass Spectrometry Analysis of Bee Bread

The presence of phenolic compounds was assessed by liquid chromatography-mass spectrometry. Based on studies conducted by Isidorov et al. [10] and Urcan et al. [21], bee bread was screened for the presence of fourteen phenolic compounds, which included apigenin, benzaldehyde, caffeic acid, chrysin, ferulic acid, gallic acid, hydroquinone, isorhamnetin, kaempferol, mangiferin, naringenin, p-coumaric acid, quercetin, and resveratrol. Briefly, ten gram of powdered bee bread was soaked in 5 mL of methanol and sonicated (Lab Companion, Model UC-20, Seoul, Korea) for 30 min. The solution was filtered and evaporated using rotovap (Buchi, Rotavapor^®^ R-300 system, Flawil, Switzerland) to make a stock solution of 3 mg/mL. The resulting solution was filtered through a membrane filter (pore size 0.22 µm) before analysis. The sample was analysed by LTQ Orbitrap mass spectrometer (Thermo Scientific, San Jose, CA, USA). Acetonitrile and 0.1% formic acid were used as the mobile phase. The spectral *m*/*z* from 100–1000 was recorded in positive ionization mode. The mass spectrophotometry was performed in electrospray ionisation conditions and positive mode with the following parameter settings: source accelerating voltage = 4.0 kV; capillary temperature = 280 °C; sheath gas flow = 40 arb; auxiliary gas = 20 arb.

### 2.4. Animals and Diet

Thirty-two male Sprague Dawley rats of age between 8 and 10 weeks (180–230 g) were obtained from Animal and Research Centre (ARASC), Universiti Sains Malaysia, Kubang Kerian, Kelantan. Animals were housed in an individual cage with a constant temperature at 22–24 °C, given a 12 h light and dark cycle, and supplied with normal rat chow pellet with water ad libitum. Animals were handled according to guidelines provided from local Ethics Committee (USM/Animal Ethics Approval/2016/(98) (744)). Following an acclimatization period, rats were administered either a normal diet or high-fat diet (HFD). The normal diet is a standard Altromin pellet imported from Germany by Sterling Ascent, Malaysia. Obesity was established by feeding with a HFD using the previous method with slight modifications, consisting of 32 g of ghee (saturated fat from animal), 68 g of powdered normal rat chow, 300 mg of calcium, 100 UI of vitamin D3, and 12% cholesterol powder [22]. After a dough-like consistency formed, foods were shaped into small hand-balls and kept at 4 °C overnight to feed the rats in the next morning. Foods were prepared every two days to avoid lipid oxidation. The nutrient composition of normal and HFD is shown in Table 1.

### 2.5. Experimental Design

A pilot study was conducted with three different doses of bee bread (0.5, 1.0, and 1.5 g/kg/day) (*n* = 3/group) administered for 6 weeks via oral gavage to determine the best dose of bee bread in HFD-induced obese rats. According to Reagen-Shaw et al. [23], the lowest dose, i.e., 0.5 g/kg, was calculated based on the body surface area normalization method, relative to the local human consumption of bee bread, which is 5 g/day. Bee bread at the dose of 0.5 g/kg/day was chosen as the best dose and used in the present study, as it reduced Lee obesity index, TC, and LDL levels in HFD-induced obese rats (unpublished observation). 

Thirty-two male Sprague Dawley rats were randomly divided into four groups (*n* = 8/group); i.e., normal group (N, on normal rat chow pellet and distilled water), high-fat diet (HFD, on high-fat diet and distilled water), HFD + BB (on high-fat diet and bee bread at 0.5 g/kg/day), and HFD + O (on high-fat diet and orlistat at 10 mg/kg/day). Normal rat chow pellets and the HFD were given ad libitum. Distilled water, bee bread, and orlistat were administered to rats via oral gavage for 6 weeks. Body weight and food intake were measured every other day. At the end of experimental period, Lee obesity index was calculated using a previous method [24], and a value of less than 315 was considered as normal [25]. Animals were sacrificed after being anaesthetised with ketamine 90 mg/kg and xylazine 5 mg/kg. Blood was collected from posterior vena cava for serum biological markers. Thoracic aorta was immediately excised, rinsed in ice-cold phosphate buffer solution, and homogenized for assessment on the levels of oxidant-antioxidant markers and fatty acid synthase (FAS) activity. Section of aortic arch was analysed for the presence of atherosclerotic plaque. Adipose tissue was dissected out and stored in 10% formalin for histological study.

### 2.6. Measurement of Lipid Profile and Atherogenic Index

Total cholesterol was determined by Architect c total cholesterol kit (ARCHITECT c cholesterol kit, Abbott, IL, USA) using an enzymatic-colorimetric method, which produced the end product quinoneimine from hydrogen peroxide (coefficient of variation, CV ≤ 3% and sensitivity 18.26 mmol/L). TG was determined using Architect c triglyceride kit (ARCHITECT c Triglyceride Reagent kit, Abbott, IL, USA), which hydrolysed lipase to free fatty acids and glycerol (CV ≤ 5% and sensitivity 16.05 mmol/L). Reaction changes for TC and TG were measured at 500 nm (ARCHITECT c System, Abbott, IL, USA). High-density lipoprotein was measured based on elimination of chylomicrons, LDL, and very-low density lipoprotein by cholesterol esterase, cholesterol oxidase, and catalase using Biosino Direct HDL-Cholesterol reagent kit, Biosino Bio-Technology and Science Inc, Beijing, China (sensitivity up to 2.586 mmol/L). Absorbance value was determined at 600 nm (ARCHITECT c System, Abbott, IL, USA). LDL was determined by the formula described by Friedewald et al. [26]: LDL (mmol/L) = (TC − HDL − TG)/5. Atherogenic index was calculated using formula: AI = (TC − HDL-C)/HDL-C [27].

### 2.7. Determination of Aortic Oxidant/Antioxidant Status Markers and Fatty Acid Synthase (FAS)

Aortic tissue was homogenized using a tissue grinder (Tissue Grinder G50, Coyote Bioscience Co., Ltd., Beijing, China) in an ice-chilled 0.1 M phosphate buffer solution, pH 7.4 and centrifuged (Avanti J-HC, Beckman Coulter, IN, USA) at 4000 rpm for 15 min. Supernatant was collected and used to analyse oxidant-antioxidant markers and FAS using procedures described by respective experimental protocols. Aortic oxidised LDL (oxLDL) and MDA were determined by commercially available kits from Northwest (Vancouver, WA, USA) and Cloud-Clone (Houstan, TX, USA), respectively. Aortic antioxidant enzymes such as superoxide dismutase (SOD), glutathione peroxidase (GPx), and catalase (CAT) were determined by commercially available kits from Bioassay (San Francisco, CA, USA). Level of FAS activity was determined using a commercially available kit (Cloud-Clone, Houstan, TX, USA). 

### 2.8. Assessment on the Presence of Atherosclerotic Plaque in Aortic Arch

The aortic arch was transversely cut at about 2 mm from, where it emerged in 2 cm length. Clean aortas were fixed with 78% methanol followed by incubation with Oil Red O solution for 50–60 min. Aortas were washed twice with 78% methanol followed by phosphate buffer solution. En face images of aortic arches were visualized under a stereomicroscope (Olympus SZ, OLYMPUS, Tokyo, Japan) at ×20 magnification [28].

### 2.9. Histology of Adipose Tissue

Adipose tissue was processed and embedded in paraffin. A sample block was cut into sections of 5 µm size and fixed on glass slides. After drying, all slides were stained with haematoxylin and eosin (H&E) and inspected under a light microscope (Leica DM750, LEICA, Wetzlar, Germany).

### 2.10. Statistical Analysis

Data analysis was carried out using Statistical Package of Social Science (SPSS) version 22. After assessment for normal distribution and homogenous variance, the one-way analysis of variance (ANOVA) test was used and followed by Tukey’s post-hoc test for multiple comparisons. *p* ˂ 0.05 was defined as statistically significant. Values are expressed as means (with standard deviations).

## 3. Results

### 3.1. Liquid Chromatography-Mass Spectrophotometry Analysis of Bee Bread

Five phenolic compounds were identified in the bee bread used in the present study. Isorhamnetin showed highest level of mass spectrum (317.07 *m*/*z*), followed by kaempferol (287.06 *m*/*z*), apigenin (271.06 *m*/*z*), ferulic acid (195.09 *m*/*z*), and caffeic acid (181.12 *m*/*z*) (Table 2).

### 3.2. Lee Obesity Index, Weight Gain, Food, and Calorie Intake 

After 6 weeks, all rats in HFD, HFD + BB, and HFD + O groups were obese, as their Lee obesity indices were more than 315 compared to the N group. The Lee obesity index was significantly lower in the HFD + BB group compared to HFD group. There were no significant differences in weight gain and food intake among all groups. Calorie intake was significantly higher in all groups which received the HFD compared to the N group, but it was not significantly different among all groups that received the HFD (Table 3).

### 3.3. The Effects of Bee Bread on Lipid Profile and Atherogenic Index

TC and LDL levels were found to be significantly higher in HFD group compared to N group and were significantly lower in HFD + BB group compared to HFD group. No significant differences were found for TG and HDL levels among all the groups. Atherogenic index was significantly lower in the groups supplemented with bee bread (HFD + BB) and orlistat (HFD + O) compared to the HFD group. However, the indices were not significantly different between HFD + BB and HFD + O groups (Table 4).

### 3.4. The Effects of Bee Bread on Aortic Oxidant-Antioxidant Status and FAS Activity

Aortic oxLDL and MDA levels were significantly higher in the HFD group compared to the N group and significantly lower HFD + BB compared to the HFD group. OxLDL was significantly lower in the HFD + O group compared to the HFD group. GPx and CAT activities were significantly lower in the HFD group compared to the N group. Meanwhile, significant increases of SOD and GPx activities were seen in HFD + BB group when compared to the HFD group. GPx activity was significantly higher in HFD + O group compared to N, HFD, and HFD + BB groups. The HFD group had significantly more FAS compared to the N group. However, in the HFD+BB group, the FAS level was significantly lower than for the HFD group (Table 5). 

### 3.5. The Effects of Bee Bread on the Presence of Atherosclerotic Plaque

**Figure 1:**
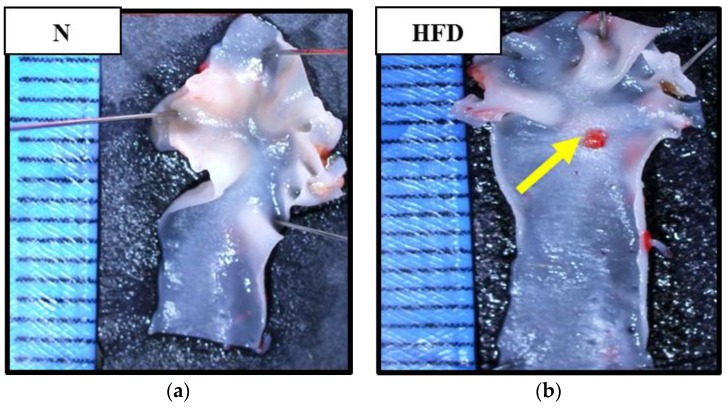

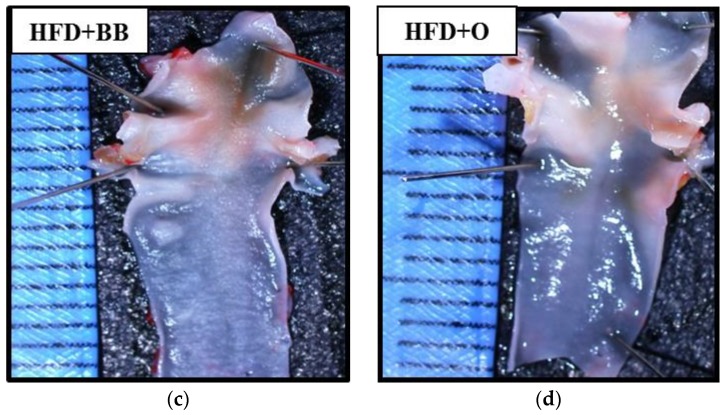
Macroscopic findings of aortic arch segment in Oil Red O staining under a stereomicroscope. Aortic arch segments from N (**a**), HFD + BB (**c**), and HFD + O (**d**) groups show intact aorta arch segments without atherosclerotic plaque. However, the segment from HFD group shows the presence of atherosclerotic plaques (yellow arrow) in the aortic arch (**b**). N, normal; HFD, high fat diet; HFD + BB, high-fat diet and 0.5 g/kg bee bread; HFD + O, high-fat diet and 10 mg/kg orlistat.

Figure 1 shows representative pictures of aortic arch segments in Oil Red O staining. Aortic arch segments from N, HFD + BB, and HFD + O groups were intact without the presence of atherosclerotic plaque. Whereas an aortic arch segment from HFD group showed a red-stained atherosclerotic plaque within the curvature of aortic arch.

### 3.6. The Effects of Bee Bread on Histology of Adipose Tissue

**Figure 2:**
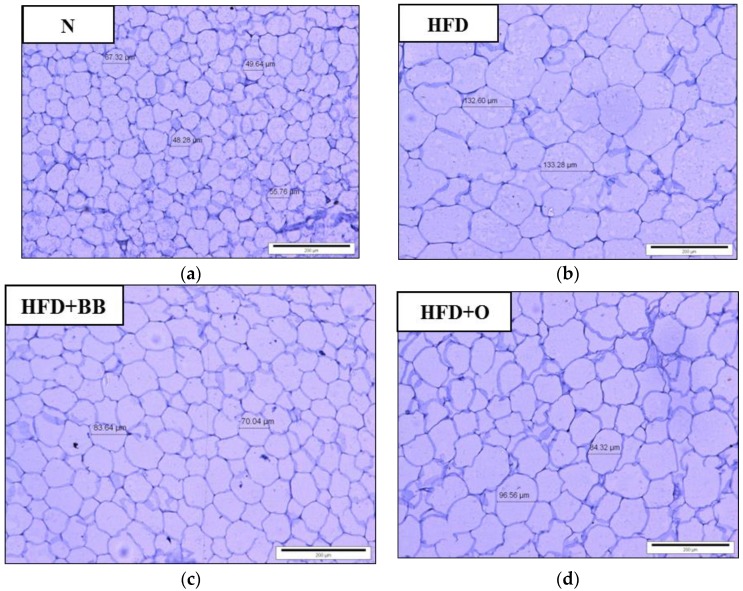
Histological findings of adipose tissue in H&E staining under light microscope. HFD group (**b**) has relatively larger adipocyte size compared to N group (**a**). Both HFD + BB (**c**) and HFD + O (**d**) groups had relatively smaller adipocyte size compared to the HFD group. N, normal; HFD, high fat diet; HFD + BB, high-fat diet and 0.5 g/kg bee bread; HFD + O, high-fat diet and 10 mg/kg orlistat. (Magnification ×200, Haematoxylin and Eosin staining).

Figure 2 shows the difference in adipocyte sizes of adipose tissue from each representative experimental group using H&E staining. Adipocyte size in HFD group was relatively larger compared to the N group. The sizes were observed to be smaller in HFD + BB and HFD + O groups compared to that of the HFD group.

## 4. Discussion

In the present study, the phenolic compounds and the anti-atherogenic effect of bee bread were evaluated in HFD-induced obese rats. After 6 weeks of continuous administration of HFD, Lee obesity index was significantly higher (more than 315) in the HFD group compared to N group, suggesting that the HFD used in the present study successfully induced an animal model of obesity. As compared to normal diet, the HFD contained higher caloric and fat contents, but lower carbohydrate and protein contents, as shown in Table 1. These differences were due to the mixture of 68 g normal diet with 32 g ghee, 300 mg calcium, 100 IU vitamin D, and 12% cholesterol powder based on the previous study [22] with slight modifications. Hence, the obesity in the HFD group could be due to high composition of fat component in HFD regime ingested, as significantly higher calorie intake was found in this group. HFD + BB group had a significant decrease in Lee obesity index when compared to HFD group, suggesting the anti-obesity effect of bee bread. Although Lee obesity index and weight gain in HFD + O group were observed to be lower compared to HFD group, the difference was not statistically significant. This may suggest bee bread at 0.5 g/kg/day has a better anti-obesity effect compared to orlistat at 10 mg/kg/day in this animal model.

Hypercholesterolemia is closely associated with pathogenesis of atherosclerosis. In the present study, the levels of TC and LDL were significantly higher in the HFD group compared to the N group, similarly to a previous study [29]. A previous study hypothesized that elevated TC and TG levels have been reported to be a crucial factor in lipoprotein metabolism, and its higher concentration was attributed to increased LDL formation and deposition, which is potently atherogenic [30]. Meanwhile, levels of TC and LDL were significantly decreased in the HFD + BB group compared to the HFD group, suggesting a hypocholesterolaemic property of bee bread. A similar finding was also found in a study in which there were significant decreases of TC and LDL levels in obese and overweight patients when combined with honey [15]. Significant low level of LDL was also observed in the HFD + O group, which is consistent with a previous study, due to its action towards inhibition of gastric and pancreatic lipase enzymes [31]. The atherogenic index values of HFD + BB and HFD + O groups were significantly lower (by 40%) than the HFD group’s, indicating that bee bread and orlistat are able to reduce the risk of CVD as high atherogenic index is an indicator of high risk to develop CVD [32]. Hence, it is plausible to suggest that both bee bread and orlistat have LDL-lowering effects which might reduce the progression of atherosclerosis.

Growing evidence supports that increased oxidative stress is attributed by the presence of excessive free radicals which interplay between hypercholesterolemia and atherosclerosis [33]. Oxidative modification of LDL plays an immense role in the initial development of atherosclerosis and promotes further accumulation of free radicals in the arterial wall. A significant increase of aortic oxLDL and MDA levels in the HFD group might suggest an excessive formation of oxidative stress in the aortic tissue following HFD ingestion as a consequence of the lipid oxidation process [34]. Meanwhile, these oxidative stress markers demonstrated significantly lower levels in the HFD + BB group compared to the HFD group. The HFD + O group had significantly lower oxLDL level compared to the HFD group, without any changes in MDA level. This might indicate that bee bread may have a greater ability to reduce lipid oxidation compared to orlistat. Antioxidants have been shown to stabilize free radicals, thereby reducing oxidative damage within biochemical, cellular and molecular levels [35]. HFD group had significantly lower levels of aortic GPx and CAT activities compared to N group which could be due to suppression or deactivation of these enzymes by oxidative stress. Significant increase of SOD and GPx activities in the HFD + BB group could be responsible for the lower oxLDL and MDA levels found in this group, which suggest the involvement of antioxidants in reducing the increase of lipid oxidation. This antioxidant effect could partly due to the presence of phenolic compounds such as isorhamnetin, apigenin, caffeic acid, ferulic acid, and kaempferol found in the bee bread which have antioxidant properties [36]. However, it is suggested to further quantify these identified phenolic compounds in future study. In addition, previous studies have reported that bee bread also contains other compounds that have antioxidant properties, such as amino acids, vitamins, and minerals [9,37,38]. As reported in our previous study, the bee bread used in the present study consists of carbohydrate (59.55%), protein (18.37%), and fat (4.51%) [38]. Hence, the antioxidant effect of bee bread found in the present study could also partly due to the interaction among these compounds, which needs further study. Orlistat has also shown to exert an antioxidant effect by evidence of significant increase in GPx activity compared to the HFD group. The previous study has shown involvement of antioxidant activity following orlistat administration in high-fed diet rats, whereby a significant increase of testicular and brain SOD activity was demonstrated [39]. High levels of oxLDL and MDA, suggesting high oxidative stress, with concomitant high TC and LDL levels, might explain the presence of atherosclerotic plaque in HFD group. Meanwhile, both HFD + BB and HFD + O groups showed an absence of atherosclerotic plaque, suggesting that supplementation of bee bread and orlistat for 6 weeks might protect against the formation of atherosclerotic plaque. Similarly, a study has reported the protective effect of orlistat in reducing the progression of atherosclerotic changes in rats fed with HFD [40].

The above results indicate that supplementation of bee bread for 6 weeks demonstrated anti-atherogenic effect as it significantly improved Lee obesity index, TC, and LDL levels; the atherogenic index; aortic oxidant-antioxidant status; and showed an absence of atherosclerotic plaque formation. To further assess the possible underlying mechanism of anti-atherogenic effect of bee bread, we also evaluated the level of aortic FAS activity. Significant low levels of TC and LDL in HFD + BB group might be related to the presence of ferulic and caffeic acids which are reported to exert hypocholesterolemic properties by significantly reducing TC, TG, and LDL levels. This, in turn, may reduce the risk of atherosclerosis [41,42]. The abundance of oxLDL in circulatory system is permeable to subendothelial layer, which further promotes inflammatory response that subsequently affects the endothelial wall integrity and whole vascular function [39]. A previous study has reported that the increase in the levels of adhesive molecules, such as intracellular and vascular cell adhesion molecule, is mediated by platelet-activating factor, which is crucial for adhesion and migration of leucocytes into subendothelium layer [43]. As a result, more leucocytes engulf the oxLDL, and subsequently form foam cells, which further triggers higher inflammation cascades for development of an atherosclerotic plaque [44]. Kaempferol, a flavonoid derivative, has been shown to exert an anti-inflammatory effect by reducing the aortic levels of inflammatory markers, intracellular and vascular cell adhesion molecule, monocyte chemotactic protein 1, and E selectin in rabbits as the model of atherosclerosis [45]. Hence, the anti-atherogenic effect of bee bread could also be attributed to the anti-inflammatory effect of kaempherol and the hypocholesterolemic effects of ferulic and caffeic acids that are present in the bee bread. In addition, it is also suggested to determine the role of inflammatory cytokines in a future study.

Higher fatty acids in circulation are associated with higher formation of polyunsaturated fatty acids mediated by FAS [46]. In the present study, significant higher aortic FAS in the HFD group, which represents higher fatty acid formation, could be attributed to high TC and LDL levels found in this group. This is further supported by the finding of larger adipocyte size observed in this group. A significant low activity of FAS in the HFD + BB group might explain the low levels of TC and LDL, and the presence of smaller adipocyte size in this group. Taken together, these findings may indicate the potential mechanism of anti-atherogenic effects of bee bread, which needs further study to determine its exact molecular mechanism of action.

## 5. Conclusions

Bee bread at 0.5 g/kg/day significantly improved Lee obesity index, TC, LDL, atherogenic index, aortic oxidative stress status (oxLDL and MDA levels), aortic antioxidant enzymes (SOD and GPx activities), and FAS level in HFD-induced obese rats. These findings were in accordance with our findings on en face aortas, which showed an absence of atherosclerotic plaque and lower size of adipocyte. Hence, it is plausible to suggest that bee bread has anti-atherogenic property, possibly partly due to the presence of phenolic compounds which have high antioxidant and hypocholesterolemic properties. However, further study is needed to investigate the exact molecular mechanisms of actions that contribute to the anti-atherogenic properties of bee bread.

## Figures and Tables

**Table 1:** Content of nutrients in normal and high-fat diets.

Nutrient Composition	Normal Diet (g/100)	High-Fat Diet (g/100 g)
Carbohydrate	64	46
Protein	24	12
Fat	12	31
Ash	6.9	3.8
Energy (kcal/100 g)	318.8	516.5

**Table 2:** Liquid chromatography-mass spectrophotometry analysis of bee bread.

Compounds	Molecular Formula	Molecular Weight (g/mol)	Mass Spectrum (*m*/*z*)	Retention Time (min)
Apigenin	C_15_H_10_O_5_	270.05	271.06	17.76
Caffeic acid	C_9_H_8_O_4_	180.16	181.12	17.37
Ferulic acid	C_10_H_10_O_4_	194.18	195.09	12.45
Isorhamnetin	C_16_H_12_O_7_	316.26	317.07	17.49
Kaempferol	C_15_H_10_O_6_	286.23	287.06	17.59

**Table 3:** The effects of bee bread on Lee’s obesity index, weight gain, food, and calorie intake.

	N	HFD	HFD + BB	HFD + O
Lee obesity index	312.37 ± 5.66	337.83 ± 13.01 ^a^	316.83 ± 7.70 ^b^	322.27 ± 13.97
Weight gain (g)	112.73 ± 42.22	123.20 ± 21.84	98.70 ± 35.11	104.73 ± 21.99
Food intake (g)	20.23 ± 3.61	17.94 ± 1.58	18.73 ± 2.82	20.90 ± 1.06
Calorie intake (kcal)	64.32 ± 11.47	92.41 ± 8.13 ^a^	96.46 ± 14.50 ^a^	107.61 ± 5.45 ^a^

Data are presented as means (with standard deviations) (*n* = 8 per group). N, normal; HFD, high fat diet; HFD + BB, high-fat diet and 0.5 g/kg bee bread; HFD + O, high-fat diet and 10 mg/kg orlistat. ^a^ *p* < 0.05 compared with N group; ^b^ *p* < 0.05 compared with the HFD group (one-way ANOVA followed by Tukey’s post-hoc test).

**Table 4:** The effects of bee bread on serum lipid profiles and the atherogenic index.

Serum Lipid Profile and Atherogenic Index	N	HFD	HFD + BB	HFD + O
TC (mmol/L)	1.77 ± 0.18	2.29 ± 0.20 ^a^	1.89 ± 0.23 ^b^	1.93 ± 0.23
TG (mmol/L)	0.79 ± 0.33	1.09 ± 0.35	0.73 ± 0.23	1.03 ± 0.36
LDL (mmol/L)	0.16 ± 0.11	0.43 ± 0.20 ^a^	0.14 ± 0.10 ^b^	0.16 ± 0.10 ^b^
HDL (mmol/L)	1.20 ± 0.15	1.29 ± 0.13	1.40 ± 0.14	1.30 ± 0.15
Atherogenic index	0.44 ± 0.02	0.55 ± 0.16	0.32 ± 0.08 ^b^	0.33 ± 0.12 ^b^

Data are presented as means (with standard deviations), *n* = 8 per group. N, normal; HFD, high fat diet; HFD + BB, high-fat diet and 0.5 g/kg bee bread; HFD + O, high-fat diet and 10 mg/kg orlistat. TC, total cholesterol; TG, triglyceride; LDL, low-density lipoprotein; HDL, high-density lipoprotein. ^a^ *p* < 0.05 compared with N group; ^b^ *p* < 0.05 compared with the HFD group (one-way ANOVA followed by Tukey’s post-hoc test).

**Table 5:** The effects of bee bread on aortic oxidant/antioxidant biomarkers and fatty acid synthase.

Oxidant-Antioxidant Status	Group			
	N	HFD	HFD + BB	HFD + O
OxLDL(pg/mL)	272.08 ± 41.73	468.23 ± 113.94 ^a^	287.19 ± 92.19 ^b^	310.83 ± 93.59 ^b^
MDA(nmol/mg protein)	0.18 ± 0.03	0.24 ± 0.05 ^a^	0.16 ± 0.02 ^b^	0.21 ± 0.05
SOD(Umg/protein)	3.29 ± 0.77	2.82 ± 0.43	3.86 ± 0.46 ^b^	3.48 ± 0.67
GPx(Umg/protein)	245.87 ± 12.74	232.28 ± 4.38 ^a^	269.23 ± 11.45 ^a,b^	311.53 ± 6.23 ^a,b,c^
CAT(Umg/protein)	1.13 ± 0.15	0.76 ± 0.42 ^a^	0.93 ± 0.63	1.08 ± 0.41
FAS (pg/mL)	763.07 ± 226.23	1580.47 ± 239.19 ^a^	754.09 ± 183.93 ^b^	831.70 ± 126.10

Data are presented as means (and standard deviation), *n* = 8 per group. N, normal; HFD, high fat diet; HFD + BB, high-fat diet and 0.5 g/kg bee bread; HFD + O, high-fat diet and 10 mg/kg orlistat. OxLDL, oxidised LDL; MDA, malondialdehyde; SOD, superoxide dismutase; GPx, glutathione peroxidase; CAT, catalase; FAS, fatty acid synthase. ^a^ *p* < 0.05 compared with N group; ^b^ *p* < 0.05 compared with HFD group; ^c^ *p* < 0.05 compared with HFD + BB group (one-way ANOVA followed by Tukey’s post-hoc test).
